# Supplementary material for: Prevalent and Drug-Resistant Phenotypes and Genotypes of Escherichia coli Isolated from Healthy Cow’s Milk of Large-Scale Dairy Farms in China
Source: Int J Mol Sci. 2025 Jan 8;26(2):454. doi: 10.3390/ijms26020454 (PMC11764516; doi:10.3390/ijms26020454)
Supplement: Supplementary file 1 [file ijms-26-00454-s001.zip › Table S1ú1⁄4S2.pdf]

Supplementary materials

Table S1 Target genes and primers used in the PCR reactions carried out in this study.

Target gene	Forward primer sequence (5′-3′)	Reverse primer sequence (5′-3′)	Product size (bp)
16S	GTTAATACCTTTGCTCATTGA	ACCAGGGTATCTAATCCTGTT	340
phoA	CGATTCTGGAAATGGCAAAAG	CGTGATCAGCGGTGACTATGAC	720
blaPSE	ATGCTTTTATATAAAATGTG	TCAGCGCGACTGTGATGTAT	914
blaSHV	CGCCGGGTTATTCTTATTTGTCGC	TCTTTCCGATGCCGCCGCCAGTCA	1016
blaTEM	TTGCTCACCCAGAAACGCTGGTG	TACGATACGGGAGGGCTTACC	708
blaCMY	TGATGCAGGAGCAGGCTATTCC	CTAACGTCATCGGGGATCTGC	323
blaCTX-M	ATGTGCAGYACCAGTAARGTKATGGM	TGGGTRAARTARGTSACCAGAAYCAGCGG	593
tetA	GTGAAACCCAACATACCCC	GAAGGCAAGCAGGATGTAG	888
tetB	AGTGGAGCGATTACAGAA	CATATGTCCTGGCGTGTCTA	158
sul1	TGGTGACGGTGTTCGGCATTC	GCGAGGGTTTCCGAGAAGGTG	789
sul2	CGGCATCGTCAACATAACC	GTGTGCGGATGAAGTCAG	722

Table S2 Criteria for determining the MIC value of *E. coli*

Name of antibacterial drugs	Drug break point standard（mg/L）		
	S	I	R
Ampicillin	≤8	16	≥32
Amoxicillin/Clavulanic acid	≤8/4	16/8	≥32/16
Cephalothin	≤2	4	≥8
Ceftiofur	≤2	4	≥8
Meropenem	≤1	2	≥4
Kanamycin	≤16	32	≥64
Gentamicin	≤2	4	≥8
Tetracycline	≤4	8	≥16
Doxycycline	≤4	8	≥16
Florfenicol	≤4	8	≥16
Polymyxin E	≤1	2	≥4
Ciprofloxacin	≤0.25	0.5	≥1
Sulfisoxazole	≤256	-	≥512
Sulfamethoxazole	≤2/38	-	≥4/76

S: Sensitive; I: Intermediary; R: Resistant; -: No inflection point determined
